# Supplementary material for: Splicing Enhancers at Intron–Exon Borders Participate in Acceptor Splice Sites Recognition
Source: Int J Mol Sci. 2020 Sep 8;21(18):6553. doi: 10.3390/ijms21186553 (PMC7554774; doi:10.3390/ijms21186553)
Supplement: Supplementary file 1 [file ijms-21-06553-s001.zip › supplementary data 1.docx]

**Supplementary data 1**


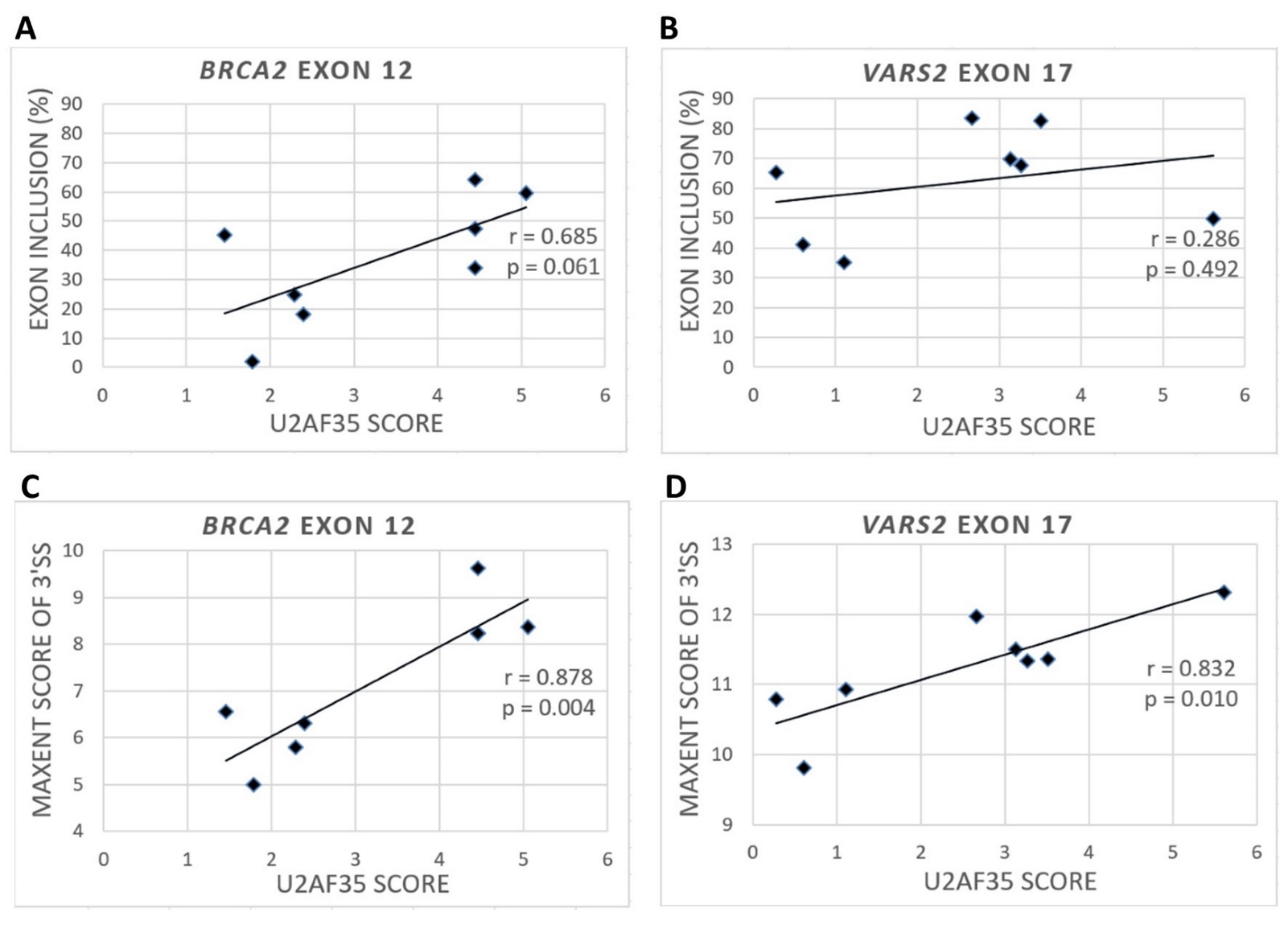


**Figure 1.** Impact of U2AF35 on both model exons. (**A, B**) Correlation between exon inclusion of *BRCA2* exon 12 (**A**) or *VARS2* exon 17 (**B**) and U2AF35 score is shown. (**C, D**) Graphs illustrating correlation between MaxEnt score and U2AF35 score in *BRCA2* exon 12 (**C**) or *VARS2* exon 17 (**D**).


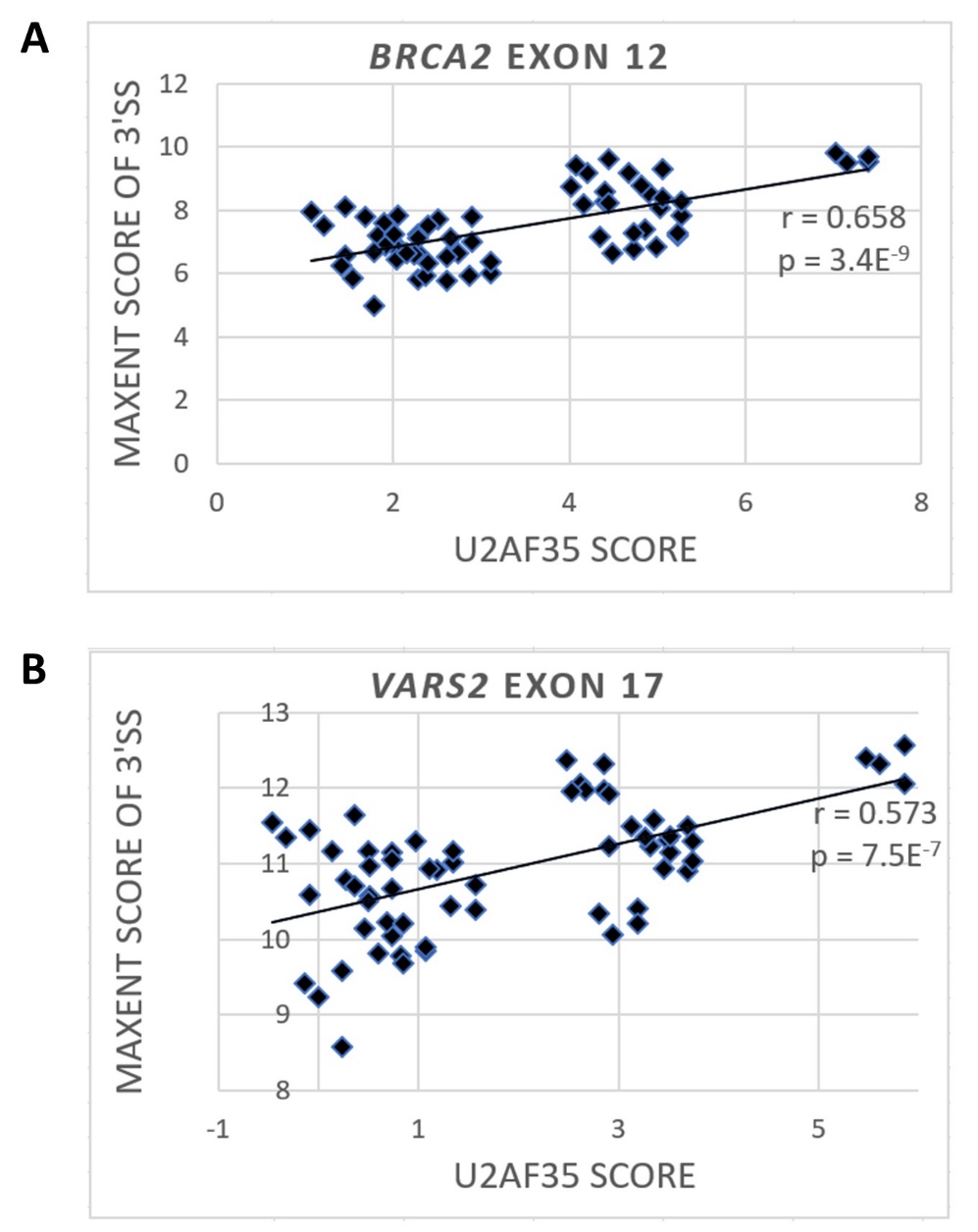


**Figure 2.** Correlation between MaxEnt and U2AF35 score in all possible exonic variants in (**A**) *BRCA2* exon 12 or (**B**) *VARS2* exon 17. **.**


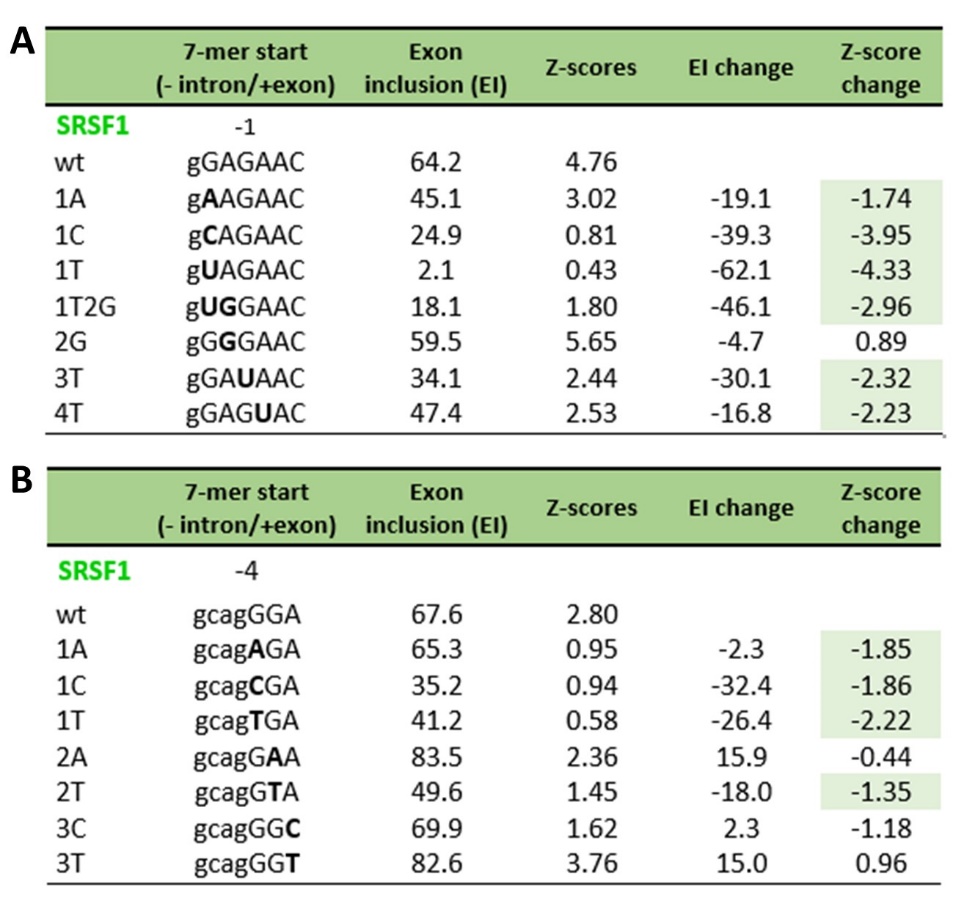


**Figure 3.** Effect of SRSF1 binding (Z-score) on exon inclusion. Z-score changes for variants of *BRCA2* (**A**) or *VARS2* (**B**) can explain splicing pattern and can represent putative disruption of binding sites. 7-mer overlapping the individual variants are shown along with their Z-scores. Value of Z-score corresponds to binding affinity of SRSF1 to each 7-mers [1]. A positive value of Z-score changes corresponds to strengthening binding site and a negative value corresponds to weakening binding site. Green boxes represent the potential ESE disruption according to EI and Z-score change.


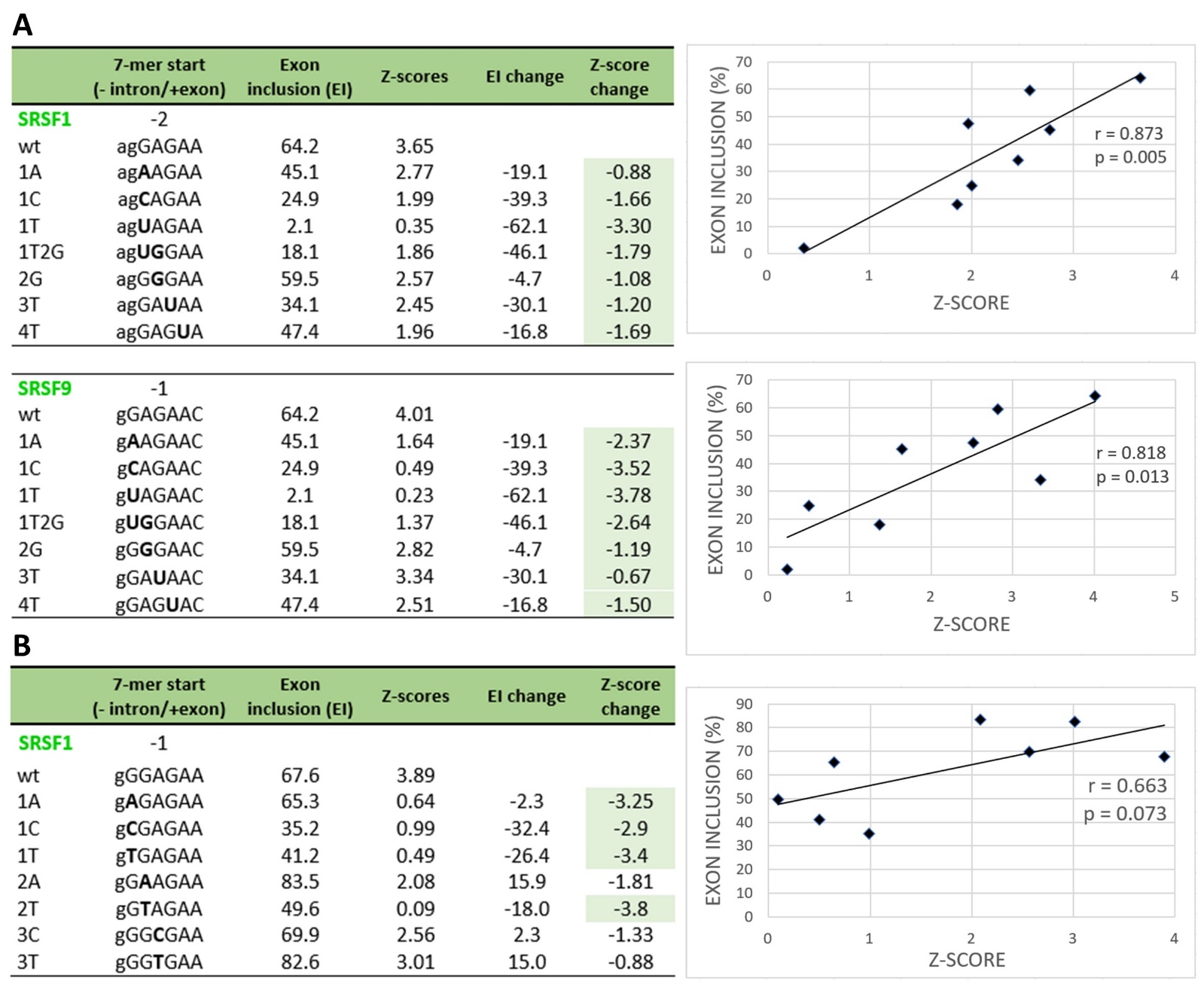


**Figure 4.** Effect of other potential binding sites. Z-score changes of putative binding site for variants of *BRCA2* exon 12 (**A**) or VARS2 exon 17 (**B**). Value of Z-scores corresponds to binding affinity of RBP to each 7-mers [1]. A positive value of Z-score changes corresponds to strengthening binding site and a negative value corresponds to weakening binding site. The green boxes corresponds to potential ESE disruption. On the right, correlation graphs with Pearson's correlation coefficient for putative RBP are shown.


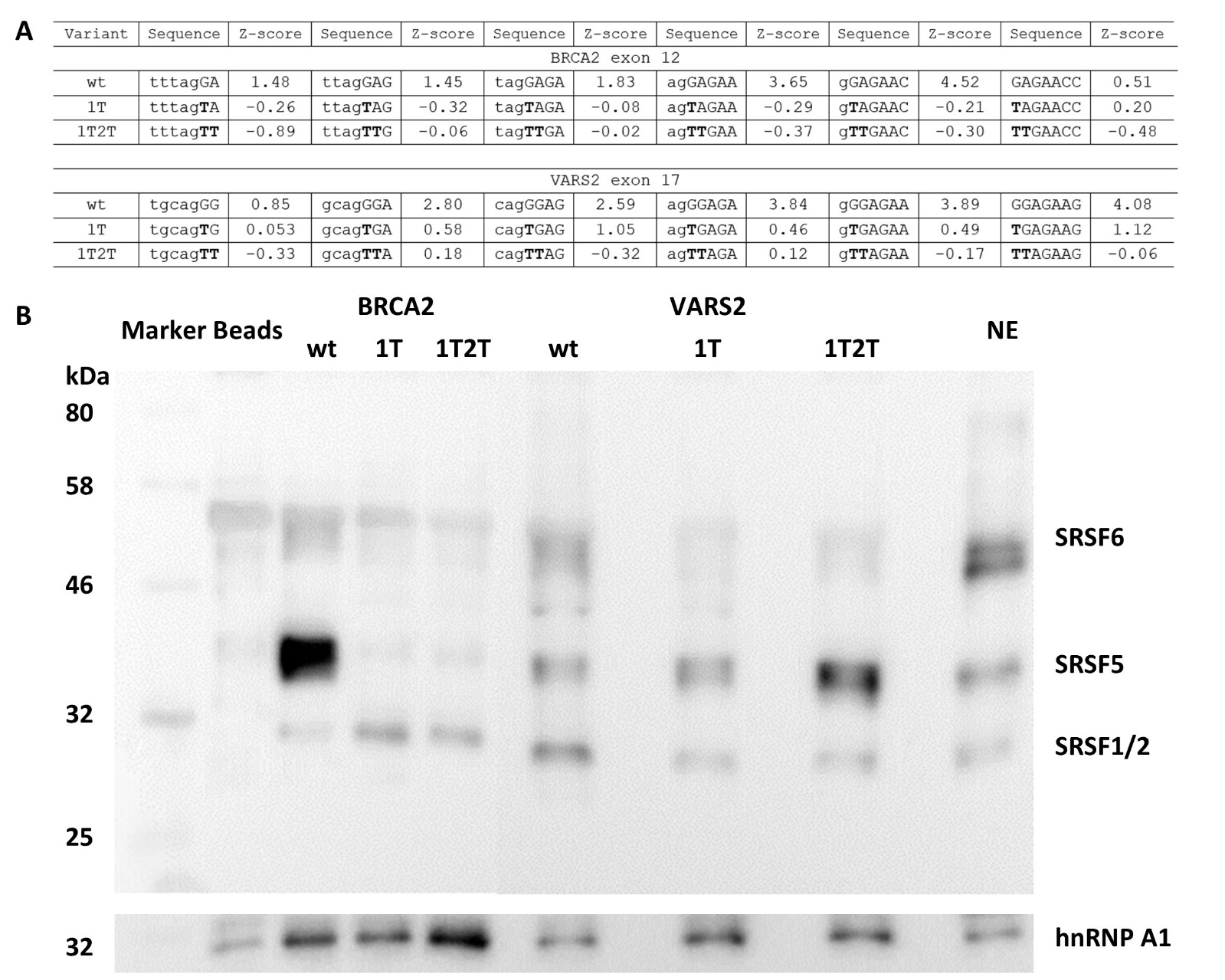


**Figure 5.** Affinity purification of RBP. (**A**) Table showing the Z-scores for all overlapping 7-mers. (**B**) Immunoblots using antibody against SR proteins (1H4; to detect SRSF1-SRSF6) and hnRNP A1 are shown.


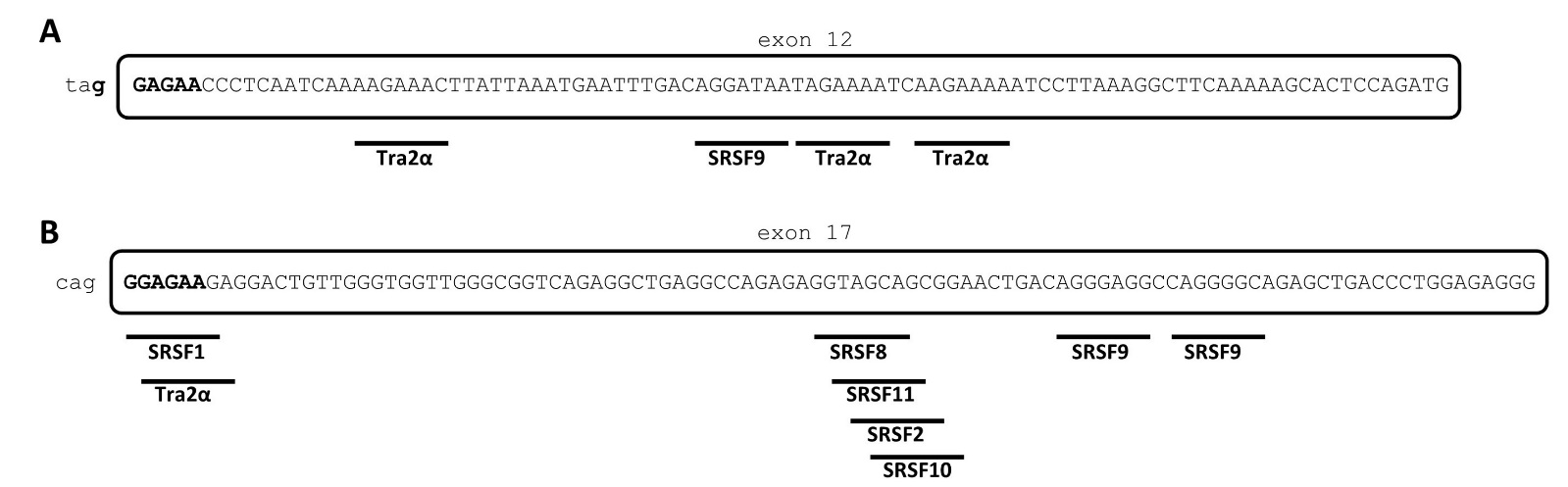


**Figure 6.** Predicted binding sites for splicing factors using oRNAment database [2] Schematic illustration of potential binding sites for different splicing regulatory proteins in *BRCA2* exon 12 (**A**) or *VARS2* exon 17 (**B**). Boxes represent exons. Uppercase letters show exonic bases, lowercase letters intronic bases and bold letters represent potential SRSF1 binding motif.


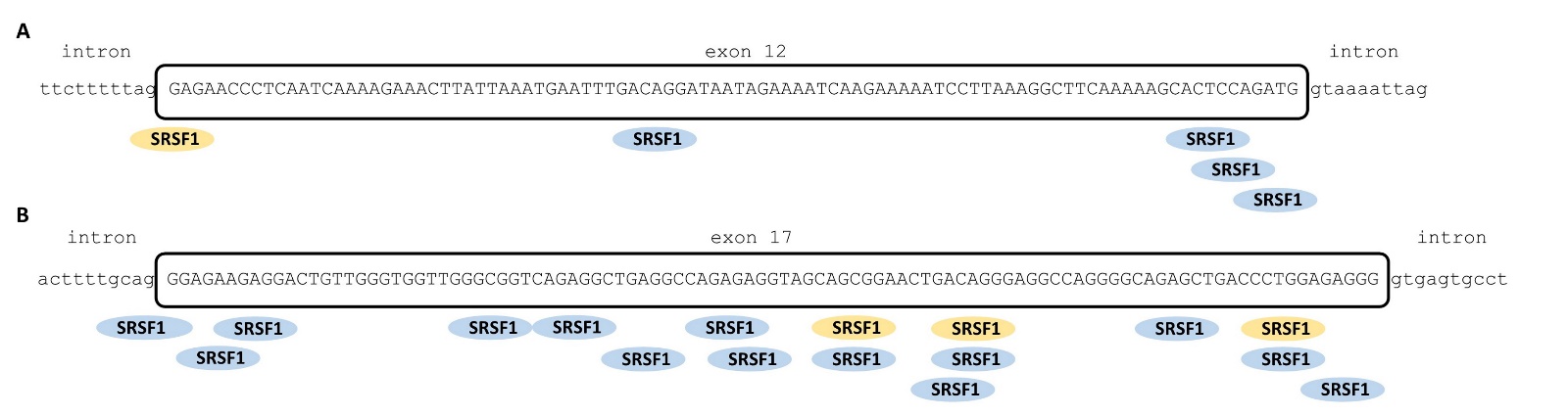


**Figure 7.** Multiple SRSF1 binding sites across exons. Schematic representation of potential SRSF1 binding sites in *BRCA2* exon 12 (**A**) or *VARS2* exon 17 (**B**) predicted by SpliceAid2 (yellow boxes) [3] or HSF (blue boxes) [4].


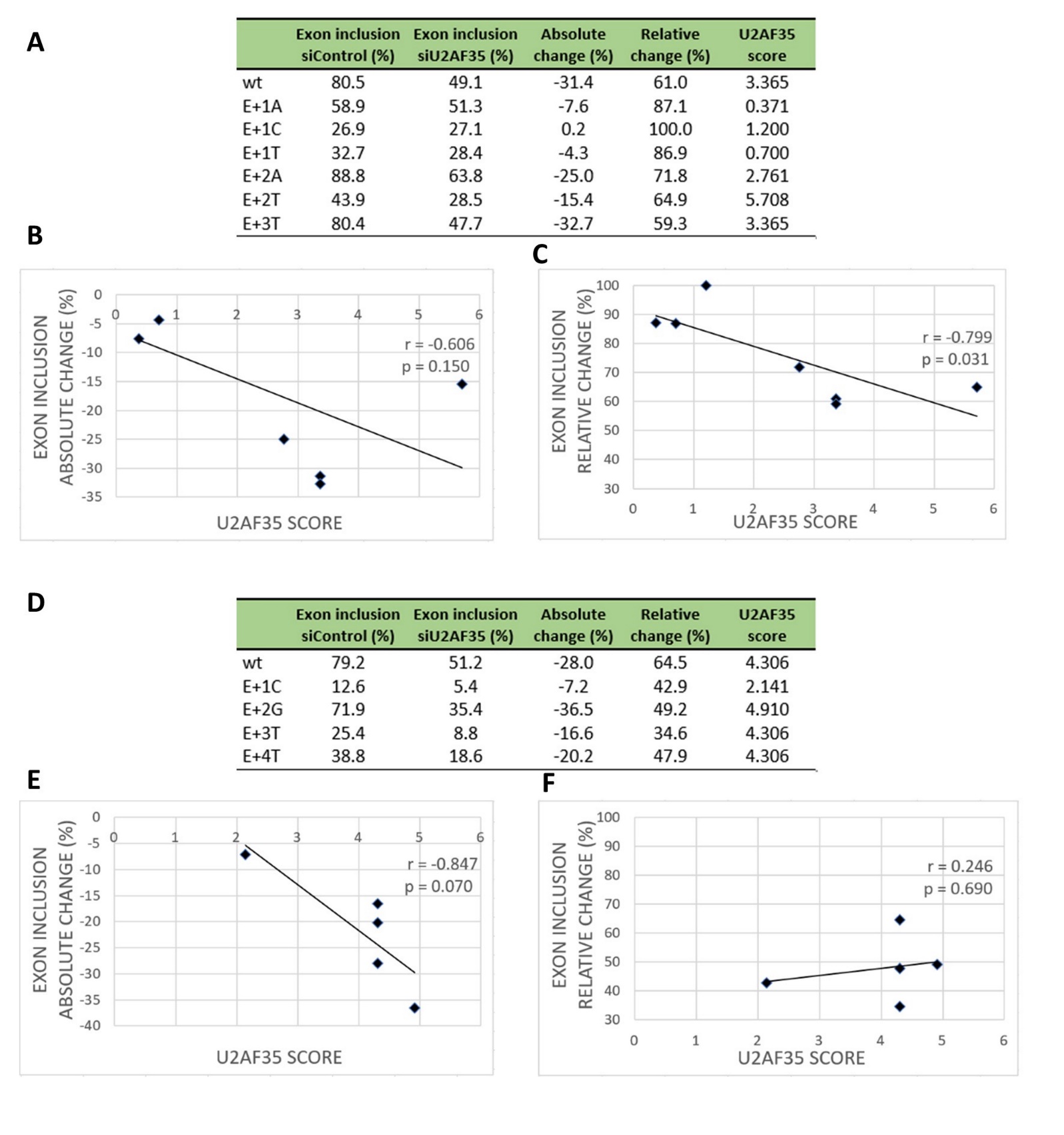


**Figure 8.** Correlation between U2AF35 knockdown and U2AF35 score. Tables and graphs showing correlation between U2AF35 knockdown and U2AF35 score in *VARS2* exon 17 (**A-C**) or *BRCA2* exon 12 (**D-F**).

**Table 1.** Summary of exons with GGAGAA motif located across intron-exon border.

**Table 2.** Correlation between the MaxEnt and U2AF35 scores in all possible variants of model exons.

**Table 3.** Complete list of Z-scores for selected RBP and Pearson´s correlation coefficients with exon inclusion.

**Table 4.** Overview of tested exons.

| **Gene** | **Exon** | **Length of upstream intron (bp)** | **Exon length (bp)** | **Length of downstream intron (bp)** |
| --- | --- | --- | --- | --- |
| *BRCA2*  *VARS2* | 12  17 | 201  231 | 96  103 | 269  223 |

| **Primer** | **5´→3´ sequence** | **Aplication** |
| --- | --- | --- |
| **Minigene** | |  |
| BRCA2_e12_f  BRCA2_e12_r | AAAAAActcgagTGGTCAAAACAGAACAAAAATG  AAAAAAggatccTCCAAATGACCAACGCATAA | cloning of *BRCA2* into pET-RIP vector |
| VARS2_e17_ f  VARS2_e17_r | CACACActcgagCAGGAGTCAGAGCTCCGCAG  CACAggatccGAAAAAGAATGGGGACAGTTGGAACCTTGGC | cloning of *VARS2* into pET-RIP vector |
| **Site-directed mutagenesis** | |  |
| BRCA2_e+1A_f  BRCA2_e+1A_r | GCCTTAAAAACATATATGAAATATTTCTTTTTAGAAGAACCCTCAATCAAAAGAAAC  GTTTCTTTTGATTGAGGGTTCTTCTAAAAAGAAATATTTCATATATGTTTTTAAGGC | preparation of *BRCA2* e+1A construct in pET-RIP vector |
| BRCA2_e+1C_f  BRCA2_e+1C_r | GCCTTAAAAACATATATGAAATATTTCTTTTTAGCAGAACCCTCAATCAAAAGAAAC  GTTTCTTTTGATTGAGGGTTCTGCTAAAAAGAAATATTTCATATATGTTTTTAAGGC | oligonucleotides to generate *BRCA2* e+1C construct |
| BRCA2_e+1T_f  BRCA2_e+1T_r | GCCTTAAAAACATATATGAAATATTTCTTTTTAGTAGAACCCTCAATCAAAAGAAAC  GTTTCTTTTGATTGAGGGTTCTACTAAAAAGAAATATTTCATATATGTTTTTAAGGC | oligonucleotides to generate *BRCA2* e+1T construct |
| BRCA2_e+1T2G_f  BRCA2_e+1T2G_r | gccttaaaaacatatatgaaatatttctttttagTGgaaccctcaatcaaaagaaac  GTTTCTTTTGATTGAGGGTTCCACTAAAAAGAAATATTTCATATATGTTTTTAAGGC | oligonucleotides to generate *BRCA2* e+1T2G construct |
| BRCA2_e+2G_f  BRCA2_e+2G_r | GCCTTAAAAACATATATGAAATATTTCTTTTTAGGGGAACCCTCAATCAAAAGAAAC  GTTTCTTTTGATTGAGGGTTCCCCTAAAAAGAAATATTTCATATATGTTTTTAAGGC | oligonucleotides to generate *BRCA2* e+2G construct |
| BRCA2_e+3T_f  BRCA2_e+3T_r | GCCTTAAAAACATATATGAAATATTTCTTTTTAGGATAACCCTCAATCAAAAGAAAC  GTTTCTTTTGATTGAGGGTTATCCTAAAAAGAAATATTTCATATATGTTTTTAAGGC | oligonucleotides to generate *BRCA2* e+3T construct |
| BRCA2_e+4T_f  BRCA2_e+4T_r | GCCTTAAAAACATATATGAAATATTTCTTTTTAGGAGTACCCTCAATCAAAAGAAAC  GTTTCTTTTGATTGAGGGTTATCCTAAAAAGAAATATTTCATATATGTTTTTAAGGC | oligonucleotides to generate *BRCA2* e+4T construct |
| VARS2_e+1A_f  VARS2_e+1A_r | CCTACTTTTGCAGAGAGAAGAGGACTG  CAGTCCTCTTCTCTCTGCAAAAGTAGG | oligonucleotides to generate *VARS2* e+1A construct |
| VARS2_e+1C_f  VARS2_e+1C_r | CCTACTTTTGCAGCGAGAAGAGGACTG  CAGTCCTCTTCTCGCTGCAAAAGTAGG | oligonucleotides to generate *VARS2* e+1C construct |
| VARS2_e+1T_f  VARS2_e+1T_r | CCTACTTTTGCAGTGAGAAGAGGACTG  CAGTCCTCTTCTCACTGCAAAAGTAGG | oligonucleotides to generate *VARS2* e+1T construct |
| VARS2_e+2A_f  VARS2_e+2A_r | CCTACTTTTGCAGGAAGAAGAGGACTG  CAGTCCTCTTCTTCCTGCAAAAGTAGG | oligonucleotides to generate *VARS2* e+2A construct |
| VARS2_e+2T_f  VARS2_e+2T_r | CCTACTTTTGCAGGTAGAAGAGGACTG  CAGTCCTCTTCTACCTGCAAAAGTAGG | oligonucleotides to generate *VARS2* e+2T construct |
| VARS2_e+3C_f  VARS2_e+3C_r | CCTACTTTTGCAGGGCGAAGAGGACTG  CAGTCCTCTTCGCCCTGCAAAAGTAGG | oligonucleotides to generate *VARS2* e+3C construct |
| VARS2_e+3T_f  VARS2_e+3T_r | CCTACTTTTGCAGGGTGAAGAGGACTG  CAGTCCTCTTCACCCTGCAAAAGTAGG | oligonucleotides to generate *VARS2* e+3T construct |
| **PCR** | |  |
| pET_f  pET_r  pET_FAM_f | CAGCACCTTTGTGGTTCTCA  GCACTGATCCACGATG  CAGCACCTTTGTGGTTCTCA-FAM | PCR primers specific for pET-RIP vector |
| **ESE-dependent splicing assay** | |  |
| pcDD_Ke1_f  pcDD_Ke1_r | AGGCTgaattcTATGGCTATGGCTATGGCTATGGCTATG GCggatccGGC  GCCggatccGCCATAGCCATAGCCATAGCCATAGCCATAgaattcAGCCT | oligonucleotides to generate Ke1 in pcDNA-Dup |
| pcDD_ggagaa_f  pcDD_ggagaa_r | AGGCTgaattcTATGGCTATGGGAGAAGCTATGGCTATGGCggatccGGC  GCCggatccGCCATAGCCATAGCTTCTCCCATAGCCATAgaattcAGCCT | oligonucleotides to generate Ke1-ggagaa in pcDNA-Dup |
| T7pro  DupS4_Seq3R  T7pro-FAM | TAATACGACTCACTATAGG  CGTGCAGCTTGTCACAGTGC  TAATACGACTCACTATAGG-FAM | PCR primers specific for pcDNA-Dup vector |
| **Pull-down assay (PD)** | | |
| PD_BRCA_wt_f  PD_BRCA_wt_f | TAATACGACTCACTATAGGGTAGGTTTAGGAGAACCCTCAA  TTGAGGGTTCTCCTAAACCTACCCTATAGTGAGTCGTATTA | probes carrying sequence of *BRCA2* wt for *in vitro* transcription (IVT) |
| PD_BRCA_1t_f  PD_BRCA_1t_r | TAATACGACTCACTATAGGGTAGGTTTAGTAGAACCCTCAA  TTGAGGGTTCTACTAAACCTACCCTATAGTGAGTCGTATTA | probes carrying sequence of *BRCA2* e+1T for IVT |
| PD_BRCA_1t2t_f  PD_BRCA_1t2t_r | TAATACGACTCACTATAGGGTAGGTTTAGTTGAACCCTCAA  TTGAGGGTTCAACTAAACCTACCCTATAGTGAGTCGTATTA | probes carrying sequence of *BRCA2* e+1T2T for IVT |
| PD_VARS_wt_f  PD_VARS_wt_r | TAATACGACTCACTATAGGGTAGGTGCAGGGAGAAGAGGAC  GTCCTCTTCTCCCTGCACCTACCCTATAGTGAGTCGTATTA | probes carrying sequence of *VARS2* wt for IVT |
| PD_VARS_1t_f  PD_VARS_1t_r | TAATACGACTCACTATAGGGTAGGTGCAGTGAGAAGAGGAC  GTCCTCTTCTCACTGCACCTACCCTATAGTGAGTCGTATTA | probes carrying sequence of *VARS2* e+1T for IVT |
| PD_VARS_1t2t_f  PD_VARS_1t2t_r | TAATACGACTCACTATAGGGTAGGTGCAGTTAGAAGAGGAC  GTCCTCTTCTAACTGCACCTACCCTATAGTGAGTCGTATTA | probes carrying sequence of *VARS2* e+1T2T for IVT |

**Table S5:** Primers, probes and oligonucleotide sequences. Lowercase letters represent restriction enzymes' sites.

**References**

1. Ray, D.; Kazan, H.; Cook, K.B.; Weirauch, M.T.; Najafabadi, H.S.; Li, X.; Gueroussov, S.; Albu, M.; Zheng, H.; Yang, A.; et al. A compendium of RNA-binding motifs for decoding gene regulation. *Nature* **2013**, *499*, 172–177, doi:10.1038/nature12311.

2. Benoit Bouvrette, L.P.; Bovaird, S.; Blanchette, M.; Lécuyer, E. oRNAment: a database of putative RNA binding protein target sites in the transcriptomes of model species. *Nucleic Acids Res.* **2020**, *48*, D166–D173, doi:10.1093/nar/gkz986.

3. Piva, F.; Giulietti, M.; Burini, A.B.; Principato, G. SpliceAid 2: A database of human splicing factors expression data and RNA target motifs. *Hum. Mutat.* **2012**, *33*, 81–85, doi:10.1002/humu.21609.

4. Desmet, F.-O.; Hamroun, D.; Lalande, M.; Collod-Béroud, G.; Claustres, M.; Béroud, C. Human Splicing Finder: an online bioinformatics tool to predict splicing signals. *Nucleic Acids Res.* **2009**, *37*, e67, doi:10.1093/nar/gkp215.
